# Eysenck’s Personality Questionnaire Scores of Heroin Addicts in Pakistan

**DOI:** 10.1192/bjo.2026.11206

**Published:** 2026-06-30

**Authors:** Mohammad Ali, Urbah Viqar

**Affiliations:** 1 Addictions Recovery Community (ARC-MK), Milton Keynes, United Kingdom; 2 Addictions Recovery Community Hillingdon (ARCH), London, United Kingdom

## Abstract

**Aims:**

To investigate the personality profiles of heroin addicts in Pakistan using the Eysenck Personality Questionnaire (EPQ) and to explore how personality traits such as extraversion, neuroticism, and psychoticism relate to substance abuse within the sociocultural and economic context of the region.

**Methods:**

A cross-sectional study in Islamabad (May 2023–May 2024) assessed 300 heroin-dependent individuals (sample size calculated using the WHO calculator), aged 18–60, in rehabilitation centres. Participants were selected using purposive sampling to meet study criteria. The Eysenck Personality Questionnaire measured extraversion, neuroticism, and psychoticism. Data were collected via self-administered questionnaires andanalysed in SPSS v29; Chi-squared tests determined significance (p < 0.05). Ethical approval was obtained from the institutional review board of Islamabad Medical and Dental College, and all participants provided informed consent.

**Results:**

Data was collected from 300 participants aged 18–60. Overall, heroin addicts had a mean extraversion score of 12.4 (SD = 4.8; range 5–20), neuroticism 17.6 (SD = 5.2; range 7–25), and psychoticism 8.3 (SD = 3.9; range 2–15), indicating average sociability, high emotional instability, and moderate aggression.

Age group differences were significant: the 18–30 group had the highest scores for extraversion (13.2), neuroticism (18.4), and psychoticism (9.1), followed by 31–45 (11.5, 17.0, 8.0) and ≥46 (11.0, 16.8, 7.5) (p = 0.02–0.04). Addiction duration was inversely related to scores: participants with <1 year of addiction scored highest (extraversion 13.5,neuroticism 19.0, psychoticism 9.5), 1–5 years scored moderately (12.0, 17.8, 8.3), and >5 years scored lowest (11.2, 16.5, 7.8) (p = 0.01–0.03).

Gender differences were not statistically significant: males had mean scores of 12.5, 17.5, and 8.4; females had 12.0, 18.0, and 8.0 for extraversion, neuroticism, and psychoticism, respectively (p > 0.05).

**Conclusion:**

Heroin addicts in Pakistan exhibit high neuroticism and moderate extraversion and psychoticism, with younger individuals and those with shorter addiction duration showing higher trait levels. These findings underscore the need for personalized interventions, such as CBT, DBT, peer support, and medication-assisted treatment, to address emotional instability and improve recovery outcomes. Policymakers should prioritize individualized treatment strategies and support further research to enhance rehabilitation effectiveness.

